# Glyphosate-Based Herbicides Alter the Reproductive Morphology of *Rosa acicularis* (Prickly Rose)

**DOI:** 10.3389/fpls.2021.698202

**Published:** 2021-06-16

**Authors:** Alexandra R. Golt, Lisa J. Wood

**Affiliations:** Department of Ecosystem Science and Management, University of Northern British Columbia Canada, Prince George, BC, Canada

**Keywords:** glyphosate, glyphosate-based herbicides, *Rosa acicularis*, reproductive deformation, pollen viability

## Abstract

Glyphosate is a broad-spectrum herbicide that is widely used in many different commercial formulations. Glyphosate-based herbicides (GBH) are used in forestry operations to reduce populations of plants that compete with merchantable conifers. Past research has found that low-dose GBH applications caused male sterility in agriculturally relevant plants, sparking a need to determine the potential impacts of forestry-related GBH applications on understory plants. We investigated the effects of GBH on the reproductive morphology of *Rosa acicularis*, a highly prevalent understory shrub within British Columbia, Canada, growing on three operational forestry cutblocks treated with 1.782 kg a.i./ha of glyphosate, in the Omineca Region, and also in a controlled experiment. We analyzed floral and pollen morphology from treated plants and compared these with untreated plants in both scenarios. Pollen viability of treated plants was reduced by an average of 66%, and >30% of anthers were non-dehiscent compared to controls across our three field sites and experimental plants. We also found alterations in pollen and petal morphology in flowers from treated sites and glyphosate residues present in floral tissues 2 years after GBH applications. It is important to fully understand how long GBH-induced change will impact forest vegetation, to preserve natural forest biodiversity and reduce anthropogenic influences on boreal forest ecosystems.

## Introduction and Background

Glyphosate, or N-(phosphonomethyl) glycine (C_3_H_8_NO_5_P), is the active ingredient of many prevalent, broad-spectrum herbicides used to reduce populations of unwanted, competitive plant species in forestry, agricultural, industrial, and domestic applications (Blackburn and Boutin, 2003; Health Canada, 2017). The majority of herbicides used in British Columbia (BC) within the past 30 years have been glyphosate-based herbicides (GBH). GBH were applied across over 600,000 hectares of forest in BC since 1987, at an average of ~17,000 ha/year since 2000 (Hunt and Matute, 2019).

A large proportion of GBH treatments occurring in BC are applied aerially (Govindarajulu, 2008; Wood, 2019). This application technique is usually prescribed in harvested forest cutblocks that have been planted with coniferous trees, such as lodgepole pine (*Pinus contorta*) and Engelmann-white spruce (*Picea glauca x engelmannii*), but where deciduous trees have grown quickly post-harvest and are out-competing the merchantable conifers. Aerial applications of GBH are used to remove this deciduous competition, but result in unintended (off-target) treatment of plants growing beneath and beside these deciduous trees; these plants often survive because they have only received a partial application. Understory plants that receive a sublethal application of GBH develop of a variety of responses, including hormesis, changes in morphology, alteration of the site of interaction, and metabolic synthesis (Belz and Duke, 2014; Sammons and Gaines, 2014; Huffman et al., 2016; Dupont et al., 2018). Glyphosate residues in plant tissues are reported to vary in persistence depending on species and plant tissue type (Feng and Thompson, 1990; Wood, 2019; Botten et al., 2021; Sesin et al., 2021).

Glyphosate is a post-emergent herbicide usually applied to the foliage of the targeted plant (Blackburn and Boutin, 2003). GBH is absorbed through the leaf cuticle and translocated by cell diffusion and vascular transport (Blackburn and Boutin, 2003). Glyphosate inhibits the enzyme 5-enolpyruvylshikimate-3-phosphate synthase (EPSPS) of the shikimate pathway, and the biosynthesis of amino acids phenylalanine, tyrosine, and tryptophan resulting in plant death when concentrations are applied as operationally recommended (Sammons and Gaines, 2014; Zabalza et al., 2017).

Glyphosate-based herbicides have been found to cause abnormal formation of reproductive structures and changes in phenology in some agriculturally relevant plants, for example, one or more morphological changes to anthers, stamen, pollen, or flowers, and decreased seed abundance in glyphosate-resistant *Gossypium hirsutum* (Pline et al., 2002; Yasuor et al., 2007), *Zea mays* (Thomas et al., 2004), *Ipomoea purpurea* (Baucom et al., 2008), and *Chenopodium album* (Boutin et al., 2014). Based on a limited pool of literature, GBH appear to have had less effect on female reproductive structures compared to male (Yasuor et al., 2007). Effects of spray drift on plant reproduction were reviewed by Cederlund (2017), who indicated that the timing of GBH application is critical in determining the severity of effect on reproduction in most species studied thus far, and pointed to a need for more research.

Despite the prevalence of GBH use, there has been very little research conducted on the morphological effects of GBH to understory plant species, especially with regard to forest plant reproductive morphology. Changes in reproductive morphology due to GBH exposure may have direct effects on pollination (Potts et al., 2010; Dupont et al., 2018). Declines in flower abundance and shifts in flowering phenology, causing a loss of floral resources, are major factors driving decline of native flower-visiting insects (Potts et al., 2010; Dupont et al., 2018). Reduced pollination success reduces fruit production and seed set, thereby reducing plant populations and food for frugivores and granivores. Most of these potential consequences, resultant from changes in reproductive morphology induced by GBH, remain unexplored or undocumented.

## Study Species

British Columbia is home to a wide variety of shrubs, one of the most prominent is *Rosa acicularis* (prickly rose). The range for *R. acicularis* stretches across North America and it is found in a wide variety of habitats from forest to rocky slopes and plains, at low to medium elevations (MacKinnon et al., 1992; Marles et al., 2012). Prickly rose grows ~1.5 m high, has stems densely covered with straight, bristly prickles and thorns, and toothed compound leaves divided into 5 to 7 leaflets, with hairy abaxial surfaces (MacKinnon et al., 1992; Young and Hawley, 2004).

The flowers of *R. acicularis* are pink and usually solitary on short side branches, forming in June and July (MacKinnon et al., 1992). Each flower has five broad petals that vary in shape from heart shaped to rounded, and there are five prominent, green sepals that are narrow, lance-like, and rounded at the base (Gray, 2011). Prickly rose has many stamens fused on the edge of the hypanthium and many carpels (Meyer, 2008). The hypanthium enlarges to become the fleshy, red, and elliptic hips, and the carpels become hard achenes (Meyer, 2008). The pollen of *R. acicularis* can be oblate-spheroidal, spheroidal, prolate, subprolate, or prolate-spheroidal, with prolate-spheroidal being the most common pollen shape (Wronska-Pilarek and Jagodzinski, 2011). Pollen of *R. acicularis* has numerous, distinct striae with narrow grooves and a polar axis with an average length of 26.5 to 37.8 μm (Wronska-Pilarek and Jagodzinski, 2011).

Prickly rose has ecological and ethnobotanical importance. For animals, it provides nutrition, cover in clear-cuts and fields, and nectar for pollinators. It has been used for many centuries by Indigenous people for medicinal and food use (Young and Hawley, 2004; Marles et al., 2012). While most parts of the plant have value, the reproductive structures of prickly rose are the most commonly used in medicines and foods. Because of its ability to colonize a variety of landscapes, *R. acicularis* is often found in and around clear-cuts and therefore is often exposed to off-target application of GBH. Since GBH are used very extensively, this research is crucial to better understand its effects on plant reproduction. Studying the effects of sublethal GBH on reproduction will inform and allow us to alter our current practices and minimize the effects of GBH on wild plants.

## Objectives and Hypotheses

The objective of this study was to quantify and compare reproductive morphology, including anther dehiscence, pollen viability and form, stigma height, petal size and shape, and flower size, in GBH-treated and control *R. acicularis* plants in operational settings and a greenhouse experiment.

We hypothesized that plants treated with GBH would have reduced pollen viability and abnormal morphology, including non-dehiscent and abnormal anthers, increased height of stigmas, smaller flowers, and abnormally shaped petals and pollen.

## Materials and Methods

### Field Sampling

**Figure 1:**
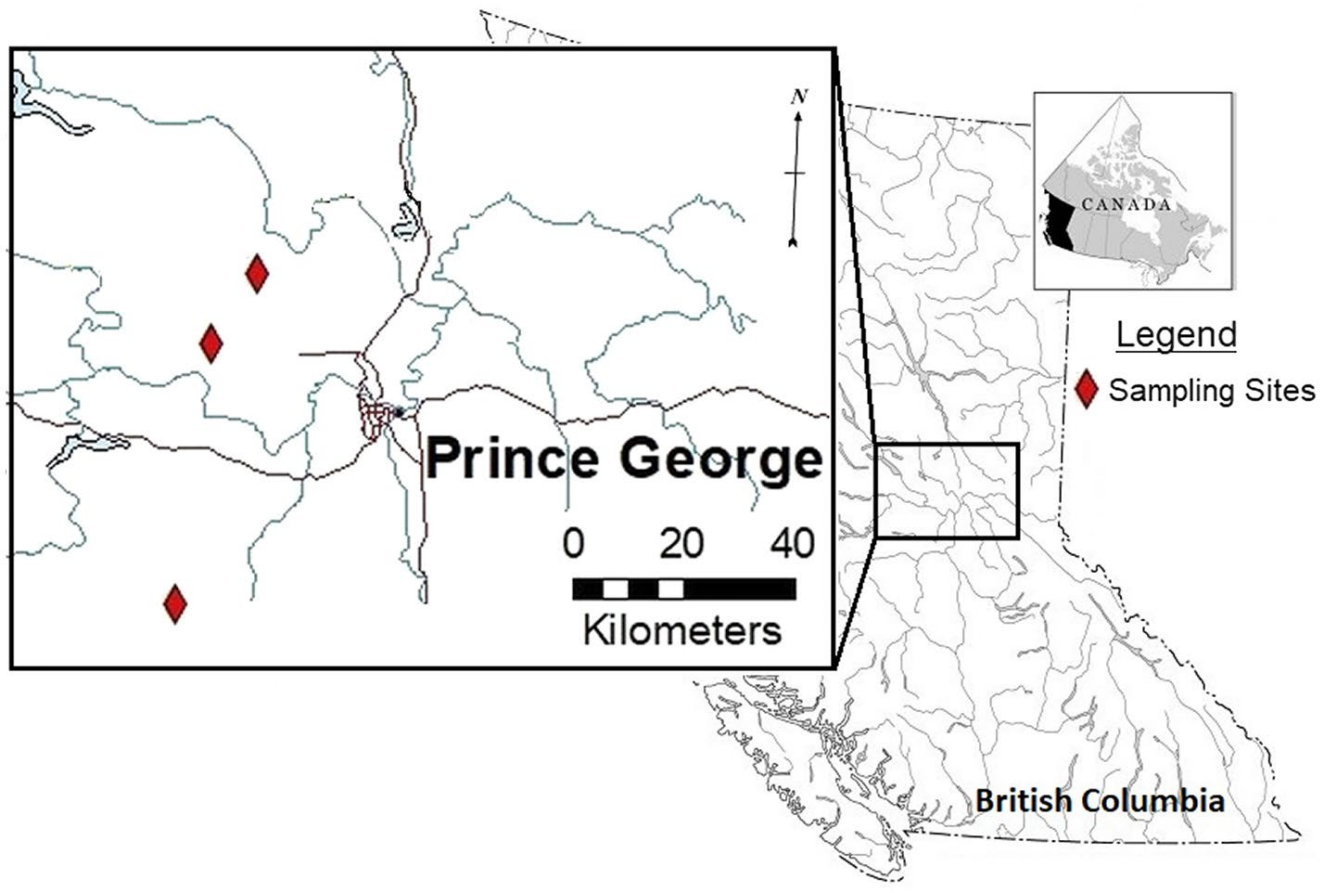
Overview of locations used for floral sampling after operational treatment of glyphosate-based herbicides (GBH) in the Omineca Region of British Columbia, Canada.

Study sites were selected within forestry cutblocks in the Omineca Region of BC, Canada (Figure 1), according to local forest industry herbicide application schedules. In August 2018, GBH product VisionMax^™^, made by Monsanto Canada Inc. (now Bayer Cropscience Inc.), was applied aerially at 3.3 L/ha using a commercial Bell 206 helicopter outfitted with a spray boom, to remove deciduous trees competing with the merchantable conifers. VisionMax^™^ contains glyphosate at a concentration of 540 g acid equivalent per liter, present as potassium salt (Bayer Cropscience Inc. 2020), and the resulting average application concentration over the cutblocks was 1.782 kg a.i./ha. Herbicide was sprayed at a constant speed and height using a 0.016 nozzle, targeting areas of the block with high aspen density as dictated by the silviculture prescription. In an effort to prevent spray drift, this application method was only permitted at wind speeds of less than 8 km per hour (Table 1; Boateng, 2002). Throughout the cutblocks, native shrubs and herbs were treated as a by-product of the aerial application technique.

**Table 1:** Forest cutblock locations treated with glyphosate-based herbicides (GBH) within in northern British Columbia, Canada, where *Rosa acicularis* was sampled for morphological testing.

Cutblock identification	Kaykay 122-3	Hoodoo 476-1	Bobtail 518-3
Latitude and longitude	54.0301°, 123.2107°	54.1051°, 123.1405°	53.5538°, 123.1854°
Total operational treatment area (ha)	16.3	56	30.8
Date of GBH application	August 23, 2018	August 22, 2018	August 30, 2018
Weather conditions at time of application	11.7^A^, 66.7^B^, 6.5^C^	16.0^A^, 69.2^B^, 0^C^	15.2^A^, 69.5^B^, 1.2^C^
Sample collection date for morphology	June 11, 2019	June 19, 2019	June 26, 2019
Sample collection date for residue testing	June 25, 2020	June 25, 2020	June 26, 2020

Weather conditions at the time of GBH application are indicated as follows:

A Average air temperature (degrees Celsius).

B Average relative humidity (%).

C Average wind speed (km/h).

Three cutblocks were selected for floral collection 1 year after GBH application (2019). Floral samples from wild prickly rose plants were collected from the following cutblocks (Table 2): the Kaykay 122-3 located northwest of Prince George, BC, the Hoodoo 476-1 located north of Prince George, and the Bobtail 518-3 located west of Prince George (Table 1; Figure 1). Treated flowers were collected from within the cutblocks, and control flowers were collected from the untreated areas outside of the cutblock at each site (Table 2). The untreated areas were selected using treatment maps and by observing the overstory plants present to confirm signs of healthy vegetation (no evidence of herbicidal application present). Treated sites were clearly identifiable by looking at the targeted trembling aspen on each site, as efficacy of the GBH product at full application concentrations is very high.

**Table 2:** Summary of *R. acicularis* flower collection, for morphological assessment in 2019, from areas treated with GBH treated and control areas 1 year prior (August 2018) in British Columbia, Canada.

Cutblock identification	Number of control individuals	Number of treated individuals	Number of control flowers	Number of treated flowers
Kaykay 122-3	5	8	14	17
Hoodoo 476-1	6	9	13	15
Bobtail 518-3	5	7	12	16

Individual plants were randomly collected over each site using an organism-to-nearest neighbor technique. After the first individual was sampled, a minimum 20 m transect was established to find the next individual to ensure individuals differed genetically. This technique was repeated until all samples were collected. A minimum of 10 treated flowers and 10 control flowers were collected per site. A minimum of 5 individual plants were sampled from at each site. Because of variability and availability of each individual, 1 to 4 flowers were collected from each individual.

Older flowers from both treated and control sites were avoided due to potential reduced pollen viability due to age. We made sure to collect mature buds (buds with sepals that have been breached and could see entire petals) as well as only recently opened flowers to try to limit exposure of the flower to environmental factors, such as light, temperature, wind, and rain. Using small gardening shears, target flowers were cut at the base of the pedicle where the herbaceous tissue met the woody tissue. Floral samples were placed in Petri dishes, sealed with parafilm, and were refrigerated until analyzed.

Additional floral samples were collected on June 25 and 26, 2020, for glyphosate residue analysis. This was completed 2 years post-spray due to the limited number of flowers available to collect during the summer of 2019. Flowers and mature buds were once again collected from the treated blocks and control areas of Kaykay, Hoodoo, and Bobtail cutblocks, using the same technique for samples collected in 2019. Flowers were grouped to form three independent composite samples from each block, composed of 60–90 individual flowers and buds. Composite samples were dried at 80°C for 24 to 48 h and then finely ground using an IKA A11 basic analytical mill. 5 g of each composite were sent to the Agriculture and Food Laboratory at the University of Guelph, Ontario, for glyphosate residue analysis by liquid chromatography with tandem mass spectrometry (LC–MS/MS); samples were run in triplicate. The glyphosate screen covers both glyphosate (parent compound) and aminomethylphosphonic acid (AMPA – major metabolite) and reports each individual component separately, if detected. An aqueous extract of a homogenized subsample of plant material was prepared, and sample extracts were acidified and separated using solid-phase extraction prior to analysis. The LC instrument employed a cation guard column for chromatographic separation (Micro-Guard Cation-H cartridge 30 × 4.6 mm), a mobile phase A (0.1% formic acid in nanopure grade H_2_O) and B (acetonitrile), with a flow rate of 1 ml/min and a total run time of 12 min. Retention times for glyphosate and AMPA were 0.9 and 4.2 min, respectively. The auto-sampler temperature was 8°C, injection volume was 50 μl, and column oven temperature was 20 ± 3°C. Validation of results was completed using a five-step detection method, to ensure no false positives. Blanks were tested along with samples to check for carry over, no co-extracting contaminants were detected, the peak detected in the samples had the same retention time for two ion transitions, the ion ratios were correct in all instances relative to the certified standard used by the laboratory, and there was consistency among sample residues found, indicating reliability.

### Greenhouse Experiment

Thirty-eight *R. acicularis* plants were collected from the forests surrounding the University of Northern British Columbia (UNBC) in Prince George, BC (Figure 1), in May 2019 and planted in 2.5 L pots with ¼ – ½ native soil, topped up with standard potting mix composed of peat, sand, perlite, vermiculite, micronutrients, slow-release fertilizer (12% N – 4% P – 8% K), and dolomite. Plants were acclimatized in the UNBC greenhouse at a daytime temperature of 22°C and a nighttime temperature of 15°C and under a natural photoperiod (12 to 16 h) throughout the 2019 growing season. Plants were watered when the surface soil was dry, and were fertilized with 20-10-20 at 200 ppm N at the beginning of the experimental period and once mid-way through the experiment. Plants were also treated with Safers insecticidal soap; 20 ml of insecticide to 1 L of water to control aphids (*Aphidoidea* sp.). Plants were placed outside in September 2019 to induce normal winter dormancy cycles and were brought back into the greenhouse on April 15, 2020. Plants began to flush on April 17, 2020. We weeded the pots to remove unwanted plant species present and topped them up with standard potting mix. Superior 70 horticultural oil, PCP 14981 (Health Canada, 2021), was applied to the plants to prevent any pest infestations.

We began to see flower buds develop on May 1, 2020. On May 6, 2020, 19 plants (half of those potted) were sprayed with VisionMax^™^ at a concentration of 0.3% using an applicator pack and using even coverage application technique, which equated to 0.45 kg a.i./ha, or approximately 20% of an operational concentration, to induce a sublethal plant response. We noticed a nutrient deficiency occurred across all pots. We analyzed pH and electrical current (EC) for the roses and found them to have a pH of 6.2 and an EC of 0.4. We applied Miracle-Gro All Purpose Shake n Feed (12-4-8) to the soil, and because EC was so low, we placed the plants on a regular weekly fertilization schedule using the irrigation fertilizer (20-10-20, 100 ppm of N) beginning May 14, 2020. We repotted the roses on June 4, 2020, into 7.57 L pots using UNBC standard potting mix and added 1 tablespoon of blood meal to each pot to aid with nutrient deficiencies. Due to regular infestation of pests, we moved the roses outside at the end of June to allow for natural predators to remove the pests.

### Measurements

Flowers were collected as they were naturally produced by the plants over the growing season. We measured each flower collected from both field and greenhouse for total floral weight, flower diameter (from petal tip to petal tip at the widest part of the flower), petal length, and petal width. The number of petals, petal shape, and petal color were also recorded. Petal color was categorized visually as: brown or white; light pink; medium pink; or dark pink. Petal shape was categorized as: All petals on the flower were heart shaped; most were heart shaped; most were round, narrow, or not heart shaped; or all were round, narrow, or not heart shaped. Flower petals were pressed in filter paper, placed in labeled Petri dishes, and refrigerated.

Male and female floral parts were separated for measurement. Twenty-five random stamen were separated from the hypanthium with a single-sided blade. Anther lengths and heights were measured using a 2 mm stage micrometer under a dissecting microscope and a digital caliper. Anther height was measured from the base of the filament where it is fused with the hypanthium to the adaxial side of the anther. Abnormal stamens were identified as stamen that were sunken, misshapen, miscolored, or not dehisced. Stamens were stored in 2.0 ml microcentrifuge tubes at 4°C. Height of the ovary was measured from the base of the ovary to the top where the filaments are fused using a digital caliper. Heights of the tallest and the shortest stigma within each flower were measured from the base of the ovary to the top of the stigma using a digital caliper.

Brewbaker and Kwack’s (B and K) medium was used to test pollen viability (Brewbaker and Kwack, 1963; Pline et al., 2003a). Pollen was deemed viable if the pollen tube formed was longer than the diameter of the grain (Pline et al., 2003a). A stock solution of B and K media was prepared by dissolving 50 mg boric acid, 150 mg calcium nitrate, 100 mg magnesium sulfate heptahydrate, and 50 mg potassium nitrate in 500 ml of deionized water. The solution was then stored at 4°C until used. The amount of sucrose needed for viability testing varies according to plant species. To determine how much sucrose was required for testing pollen viability of *R. acicularis*, some standard amounts were evaluated: 5, 10, 15, and 20% sucrose. We determined that 15% sucrose was the optimal concentration for B and K media, for *R. acicularis* (induced the highest rate of pollen tube formation). When ready to use, 15% sucrose (7.5 g) was added to 50 ml of the stock solution and stirred until dissolved.

We used freshly collected and stored pollen grains to test pollen viability and *in vitro* pollen germination. Pollen grains were collected by gently tapping the anthers on a microscope slide. Pollen was covered with a coverslip, secured with a piece of scotch tape, and stored at 4°C. We added two drops of B and K media to the slide and mixed the pollen grains into the media using a toothpick. The slides were then incubated from 24 to 36 h in a Petri dish lined with moist filter paper and sealed with parafilm to maintain humidity. Following incubation, slides were observed under an Eclipse FN1 Nikon microscope at 10× magnification. A microscope camera with NIS-Elements Imaging Software was used to view the pollen grains and capture images. We used a systematic random sampling design to determine the viability of the pollen grains of each flower from each photographed microscope frame. Images were selected in a grid-like manner across the slide for a total of 25 images per slide. From those images, pollen was counted and sorted into categories, and pollen viability was calculated.

ImageJ was used to measure the polar axis and the equatorial axis of pollen grains. Pollen was measured prior to addition of B and K reagent to compare pollen shape and size before germination. Following pollen tube germination, in instances where it was not obvious that the pollen tube was long enough for a pollen grain to be considered viable, ImageJ was used to measure pollen tube length. Pollen was classified into shape classes based on Erdtman (1943) pollen shape classification using the polar axis and equatorial axis (P/E) ratio of a pollen grain.

### Statistical Analysis

A Shapiro–Wilk test was used to test for normality throughout the data. For the most part, data were not normally distributed. Some data exhibited a skew to the left, and other data, such as polar axis length, followed a bimodal distribution. We determined significant differences in measured variables between control samples and treated samples, using Mann–Whitney *U* and Kruskal–Wallis tests, when data were continuous and nonparametric. Qualitative data of pollen shape and anther dehiscence, petal shape, and petal color were analyzed using Chi-square tests. Significant differences (*α* < 0.05) were used to ascertain what, if any, impact GBH had on the reproductive morphology of *R. acicularis*. Data were analyzed using the analysis package IBM SPSS Versions 24 and 26.

## Results

### Floral Form After Glyphosate Treatment

**Figure 2:**
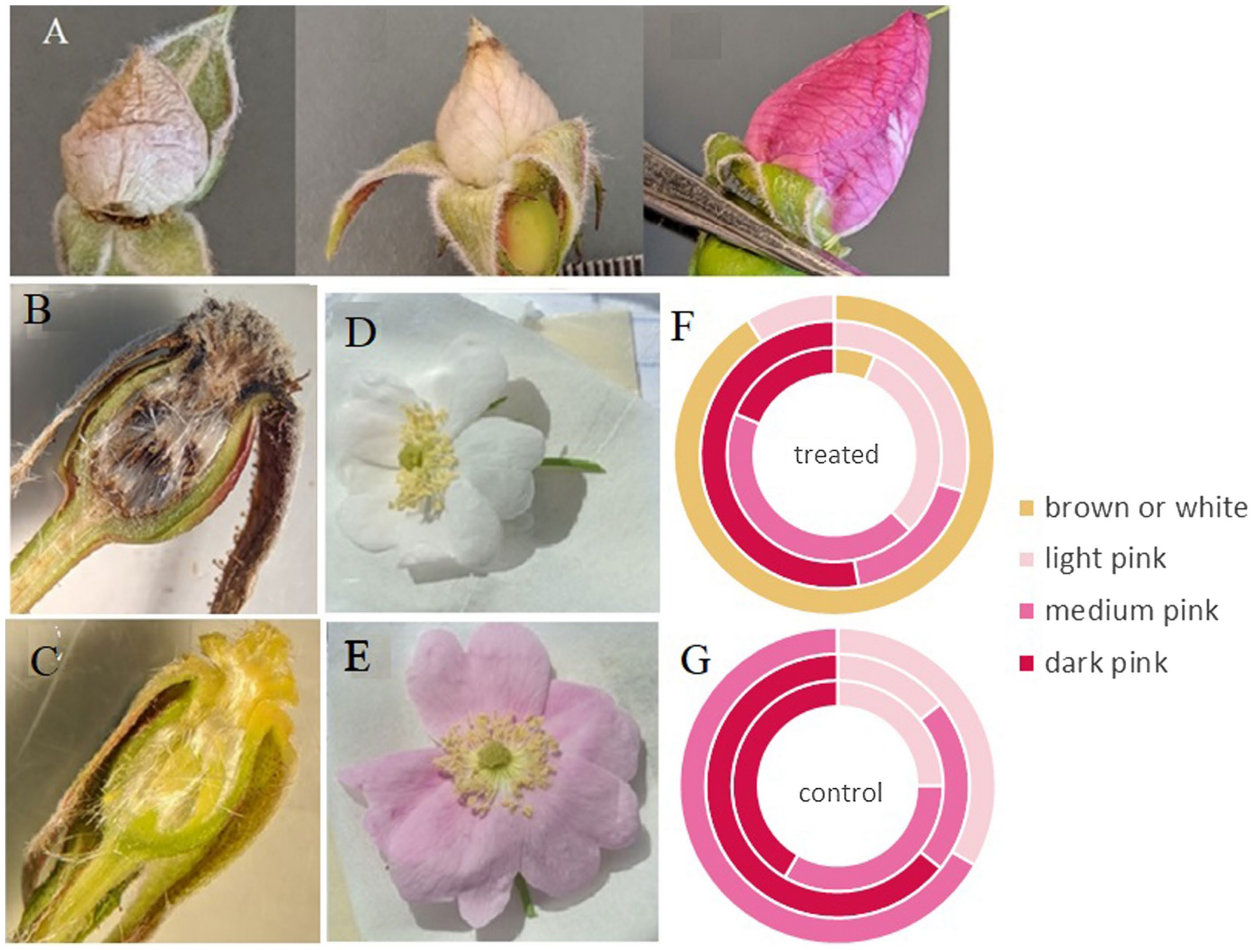
Buds and flowers of *Rosa acicularis* plants. **(A)** The range of variation in bud color and condition of those collected from operational forestry cutblocks treated with GBH one-year post-treatment. **(B)** View of inside the hypanthium in treated flowers, and **(C)** control flowers. **(D,E)** Flower color from a controlled experiment, 25% of flowers treated with GBH at 0.45 kg a.i./ha, showed white petals **(D)**, while all controls remained pink **(E)**. **(F,G)** Variation in flower color assessed in treated **(F)** and control groups **(G)** in operational forestry cutblocks sampled (outer ring = Hoodoo site, middle ring = Kaykay site, and inner ring = Bobtail site).

Petal shape and color were significantly different between treated and control flowers collected from operational cutblocks (*p* < 0.001 and *p* = 0.008, respectively). The majority of flowers collected from control areas had petals that were heart shaped (87%); however, only 12% of flowers from areas treated with GBH had heart-shaped petals (most lacked the center cleft in the petal creating a rounder petal shape). Petal color was evenly divided between white/brown, light pink, medium pinks, and dark pinks in treated areas, and in contrast, no flowers in control areas had white or brown petals and 78% of petals were categorized as medium or dark pink (Figure 2). Similarly, the treated roses in the greenhouse experiment exhibited very pale or white petals compared to the control roses (Figure 2). Furthermore, we observed that carpels in treated individuals were at times, brown in color (Figure 2). We did not see this characteristic across all treated samples, but the majority of samples within all three sites did exhibit this characteristic; this characteristic requires further investigation to quantify.

Both control and treated individuals from Hoodoo differed in some characteristics when compared to the other two sites. We found that petal length and width, ovary height, stigma heights, and stamen heights differed between controls and treated individuals at Hoodoo and were not shown to be significantly different at the other two sites. We also found that the majority of buds were smaller than buds from other sites and a greater number of flower petals were white and shriveled than at other sites (*p* < 0.001). Of note was the occurrence of individual plants on treated sites that had developed white and shriveled buds on some branches but also buds that appeared to be normal with pink petals on other branches (Figure 2).

**Figure 3:**
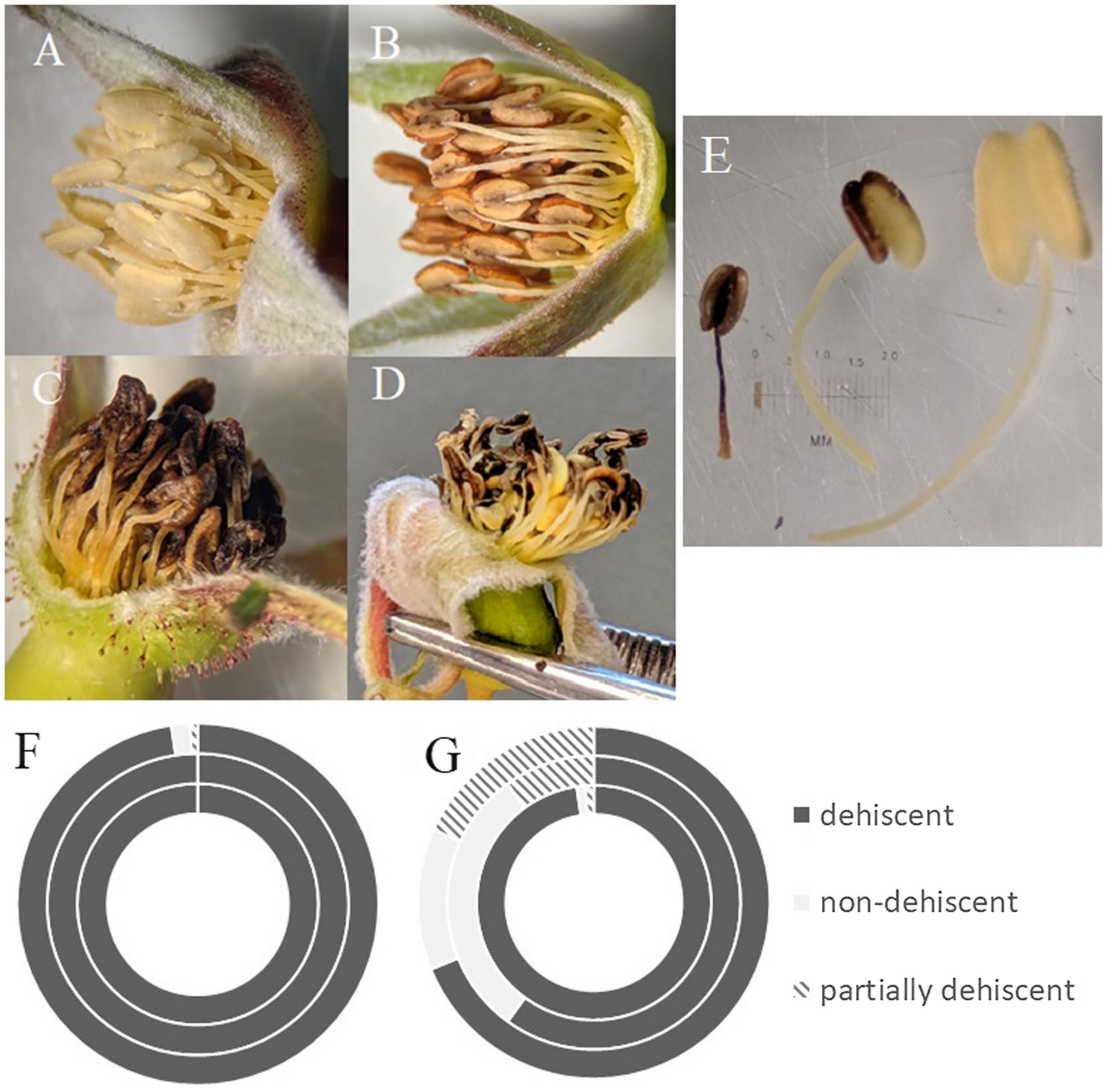
Health of *R. acicularis* floral stamen samples from three sites one year after treatment with glyphosate-based herbicide and three control sites. **(A)** Stamen from a representative control individual; **(B)** stamen found in both buds and flowers at the Kaykay forest cutblock; **(C)** stamen found at the Hoodoo forest cutblock; **(D)** stamen found at the Bobtail forest cutblock; **(E)** example of the range of variability found in stamen on treated sites; **(F,G)** the number of individuals with either dehiscent, non-dehiscent, or partially dehiscent anthers found at control sites **(F)** and treated sites **(G)** where the outer ring is the Bobtail site, the middle ring is the Hoodoo site, and the inner ring is the Kaykay site.

We found significant differences in the stamen on treated flowers from both operational areas and on treated flowers within our greenhouse experiment, versus stamen on flowers from controls. Two of the three treated cutblocks investigated contained a majority of flowers with only partially dehiscent or non-dehiscent anthers, and overall from treated areas, 66.25% of anthers were dehiscent. This is in contrast to control sites where almost all (98.66%) anthers were dehiscent (Figure 3; Table 3). This result was supported in our greenhouse experiment, where we found that all control individuals exhibited anthers that could undergo dehiscence. In our treated samples, three out of twelve individuals (25% of the sample population) had non-dehiscent anthers. We also found a significant difference in anther length between all control and treated individuals in both the operational and greenhouse settings (Table 3). On average, anthers in treated individuals were longer than anthers in the control individuals; however, the range of variability noted among the treated individuals was greater than in control plants (Figure 3). We observed differences in anther coloration between control and treated individuals in all sites. The changes we saw varied from yellow anthers with brown spotting and abnormally shaped to completely browned anthers that were sunken and non-dehiscent (Figure 3).

**Table 3:** Summary of statistics resulting from Mann–Whitney *U* and Chi-square tests conducted to determine significant differences between the characteristics of control *R. acicularis* flowers and those collected from operational forestry sites treated with GBH in northern British Columbia, Canada.

Measured variable	Mann–Whitney *U* (or Chi-square) statistic	*Z* score	Value of *p*
Floral diameter	828.00	−0.374	0.708
Petal length	619.00	−1.373	0.170
Petal width	520.00	−2.365	0.018^**^
Ovary height	916.50	−0.166	0.868
Stigma height (tallest stigma)	739.00	−1.681	0.093
Stigma height (shortest stigma)	794.50	−1.208	0.227
Anther length	579394.00	−0.385	0.700
Anther dehiscence	(1450.830)	NA	<0.001^**^
Stamen Height	535893.00	−3.371	0.001^**^
*Anther-stigma distances (four types below):*			
shortest stamen, tallest stigma	781.50	−1.319	0.187
shortest stamen, shortest stigma	907.00	−0.248	0.805
tallest stamen, shortest stigma	712.50	−1.908	**0.056**
tallest stamen, tallest stigma	824.00	−0.956	0.339
Pollen viability	209.00	−5.94	<0.001^**^
Pollen measurements (polar axis)	2390866.00	−22.88	<0.001^**^
Pollen measurements (equitorial axis)	3379849.00	−5.94	<0.001^**^
Pollen shape	(3189.580)	NA	<0.001^**^

Values considered to be significantly different are represented with

** as well as bolded.

### Pollen Form After Glyphosate Treatment

**Figure 4:**
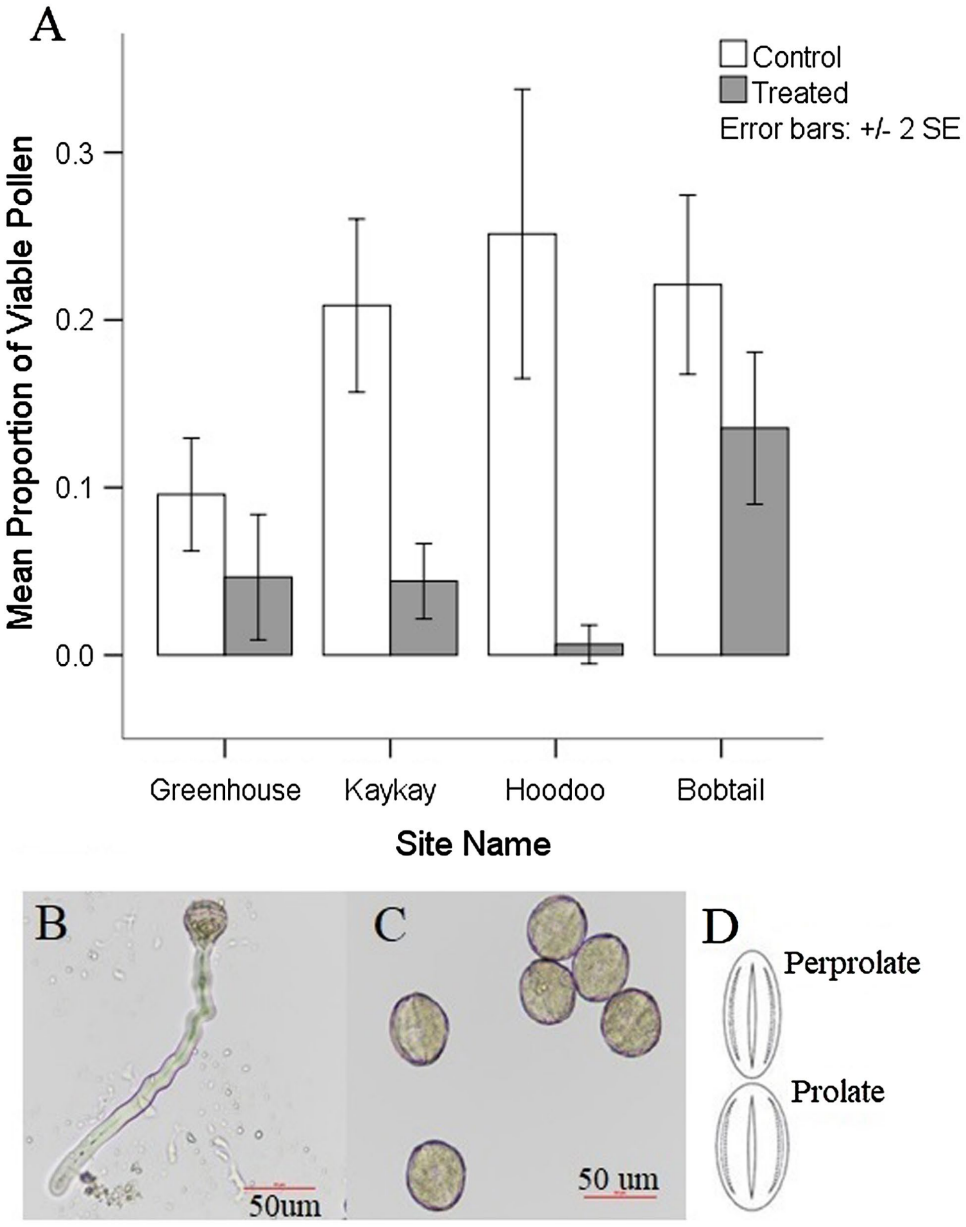
**(A)** Average pollen viability present in *R. acicularis* flowers collected from operational forestry cutblocks treated with glyphosate-based herbicides and control areas sampled in northern BC, Canada, one-year post-glyphosate treatment, as well as the pollen viability from a controlled greenhouse experiment with roses treated at 0.45 kg a.i./ha. Standard error is represented. **(B)** Viable rose pollen from a control individual undergoing germination (formation of the pollen tube). **(C)** Nonviable pollen from a treated individual that did not produce pollen tubes after exposed to germination media. **(D)** Most common shapes for pollen grains found on *R. acicularis* in operational and experimental sampling in northern British Columbia, Canada (original figure in Wronska-Pilarek and Jagodzinski, 2011).

We found a significant reduction in pollen viability at all treated sites when compared to their control sites (Table 3; Figure 4). Pollen viability at Kaykay, Hoodoo, and Bobtail was 78, 97, and 39% lower than the control samples, respectively. We also found a significant difference in pollen viability between control and treated individuals in our greenhouse experiment (Table 4), which supports the findings from our operational samples; pollen from treated individuals was 51% less viable than control samples (Figure 4). Pollen collected from the greenhouse was also significantly less viable than that collected from the operational control areas (*p* = 0.005). Furthermore, the pollen collected from one treated site was more viable, on average, than the pollen collected from the control samples in the greenhouse.

**Table 4:** Summary of values of *p* resulting from Mann–Whitney *U* and Chi-square tests conducted to determine significant differences between the characteristics of control *R. acicularis* flowers and those treated with 0.45 kg a.i./ha of GBH in a controlled greenhouse experiment.

Measured variable	Mann–Whitney *U* (or Chi-square) statistic	*Z* score	Value of *p*
Petal length	28.00	−0.771	0.481
Petal width	29.50	−0.626	0.541
Ovary height	32.00	−1.234	0.238
Stigma height (tallest stigma)	38.00	−0.772	0.473
Stigma height (shortest stigma)	30.00	−1.389	0.181
Anther length	24361.50	−3.565	<0.001^**^
Anther dehiscence	(624956.251)	NA	<0.001^**^
Stamen height	28138.00	−1.176	0.239
*Anther-stigma distances (four types below):*			
shortest stamen, shortest stigma	46.00	−0.154	0.910
shortest stamen, tallest stigma	47.00	−0.077	0.970
tallest stamen, shortest stigma	48.00	0.000	1.000
tallest stamen, tallest stigma	46.00	−0.154	0.910
Pollen viability	19.00	−2.638	0.007^**^
Pollen measurements (polar axis)	223637.50	−1.992	0.046^**^
Pollen measurements (equitorial axis)	232492.00	−0.797	0.425
Pollen shape	(998.112)	NA	<0.001^**^

Values considered to be significantly different are represented with

** as well as bolded.

We found pollen shapes at treated sites that were not observed among controls. At the control sites, we found that the most common pollen shape was perprolate (50.44% of grains) and prolate (48.42% of grains) which was also reflected in our greenhouse experiment (Figure 4). There was a similar trend seen in the treated individuals, with perprolate (38.20% of grains) and prolate (45.93% of grains) being the most common pollen shapes. However, other shapes were also present in pollen from treated sites; overall, 7.22% of the pollen grains were underdeveloped and 2.73% were abnormal to some degree, yielding unique and previously undocumented shapes. Most individuals from treated sites had wider variation in pollen shapes than seen in the controls. In the greenhouse experiment, we found no significant difference in pollen shape between control and treated individuals; the abnormal and underdeveloped grains were not observed.

Pollen grains treated with GBH were significantly smaller than controls. The polar axis ranged from 28.583 to 59.451 μm at control sites and from 21.315 to 58.823 μm at treated operational forest sites. Notably, we add to descriptive literature on pollen morphology of the genus *Rosa*, by extending the polar axis dimension, over what is previously described for *R. acicularis* (Wronska-Pilarek and Jagodzinski, 2011) up to 59.451 μm in our control samples. The equatorial axis also ranged from 14.462 to 41.420 μm at control sites and from 12.457 to 38.948 μm at treated sites. Our greenhouse experiment supported the change in pollen polar axis length when plants were treated with GBH, but no difference was observed in pollen equatorial axes in the experiment.

### Glyphosate Residue Analysis

**Figure 5:**
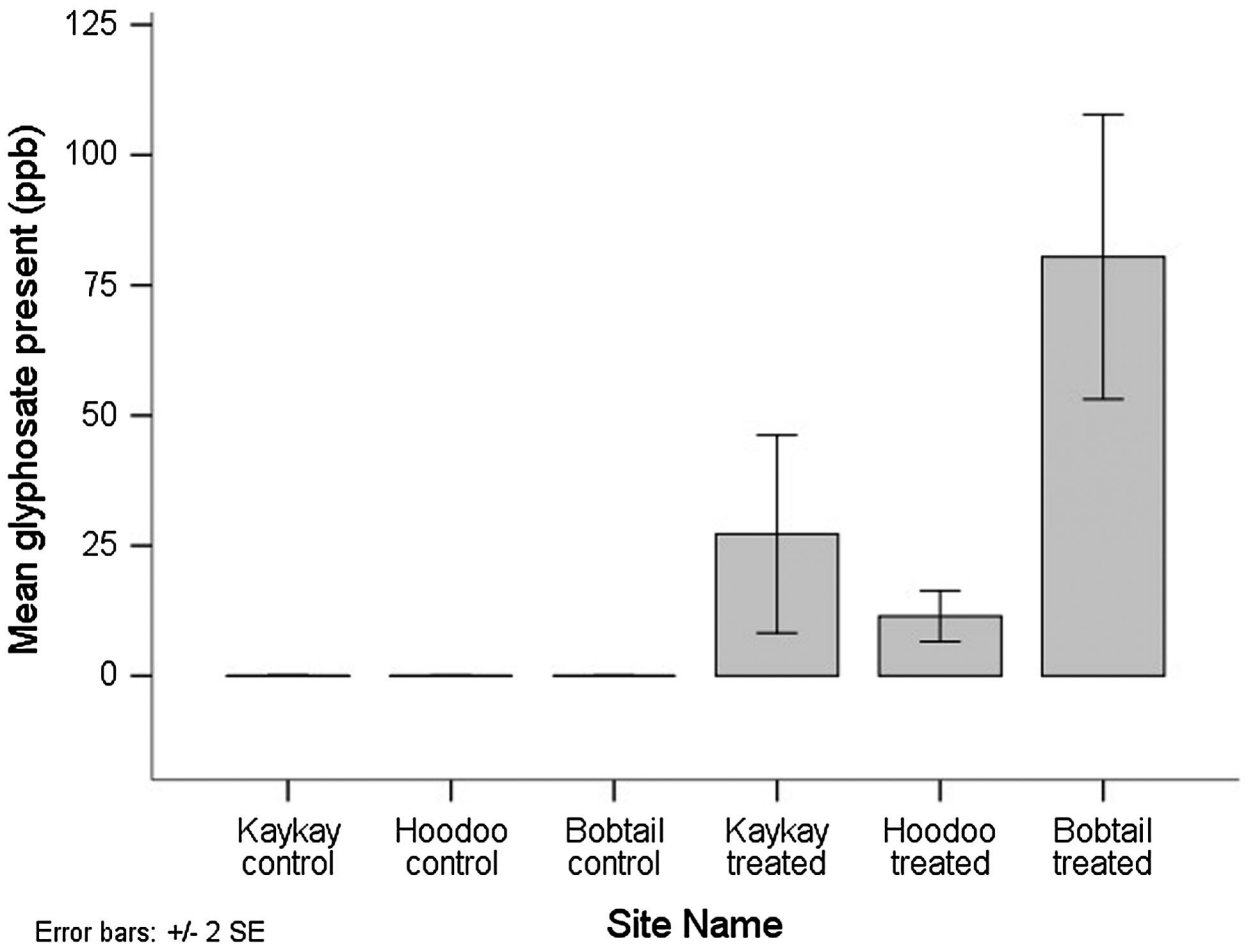
The average amount of glyphosate residue present in *R. acicularis* flowers collected from operational forestry cutblocks 2 years after they were treated with GBH in northern BC, Canada. Standard errors are represented.

All of the composite floral samples tested for glyphosate residues two years after applications, from the treated operational cutblocks, contained the residue. AMPA was not detected in any of the samples. Out of the 9 composite samples analyzed from the treated cutblocks (each composed of between 60 and 90 individual flowers), 6 samples contained residues above the minimum quantifiable limit (MQL), while in 3 samples, the presence of the compound was confirmed by LC–MS/MS, but at less than the defined quantification limit for that compound using this method. The MQL for glyphosate and AMPA was 30 ppb. When a sample falls above the minimum detection limit (MDL) of 8 ppb but below the MQL, we used a value of 9 ppb to avoid overestimation of the residue amount present. The mean glyphosate residue level detected across all treated sites was 39.7 ppb, and the maximum level detected was 110 ppb. We did not quantify any glyphosate in the control samples, although one sample did show screen positively but below the MDL of 8 ppb, and therefore, there was a significant difference in the amount of glyphosate present between control and treated samples (*p* < 0.001). Of note is that the one control sample screening positive for glyphosate (despite below MDL) was from the control site adjacent to the Kaykay block. The Kaykay block was sprayed on a day where wind speeds reached an average of 6.5 km/h at the time of application (Table 1), a speed that is very near the maximum allowable (8 km/h) for this application type, for risk of excessive spray drift. We also found that there was more glyphosate residue present in samples from the Bobtail forestry cutblock compared to the other treated sites; however, this difference was not significant (*p* = 0.282; Figure 5).

## Discussion

Our findings support the theory that male reproductive structures in plants are more sensitive to environmental factors than female reproductive structures (Pline et al., 2002; Yasuor et al., 2007). We found changes to flowers after GBH treatment, which centered largely around the male floral parts, including changes in anther dehiscence, pollen size, and pollen viability, although we also found changes to petal color and shape. The color changes seen in the petals and anthers of treated flowers could have implications on the biocommunication between pollinators and plants. Changes in coloration could indicate a change in the fluorescence emitted by the stigma, anthers, and potentially pollen. Fluorescence, especially in male floral parts such as anthers and pollen, attracts pollinators, serving as an indicator of a food source (Fukui et al., 2017), and honeybees, *Apis mellifera*, for example, can discriminate between colors and prefer blue fluorescence around 410–420 nm (Giura et al., 1995; Mori et al., 2018). Anthers and pollen emit bright blue fluorescence under UV-A radiation and create contrast with the surrounding petals thereby attracting pollinators, such as honeybees. Abnormalities in the color of these tissues could influence detection by pollinators (Mori et al., 2018).

Changes in anther dehiscence and pollen viability, combined with reduced biocommunication, may lead to a reduction in the success of pollination. Anthers that do not undergo dehiscence will not be able to release pollen, thereby rendering the male parts sterile (Yasuor et al., 2007). Failure of the pollen grain to germinate, reducing pollen viability, may also reduce the success of pollination and subsequent fertilization of ovules within the carpels. Fruit and seed set depend on the ability of the pollen grain to grow a pollen tube and successfully fertilize the ovules. Ultimately, these compounding morphological changes and interruptions in ecological interactions between species could decrease fruit production, further illustrating the influence that small changes in plant morphology may have on a larger trophic system.

Since glyphosate is a desiccation agent (Griffin et al., 2010), it follows that some of the deformations observed in morphology could be due to the influence of GBH as it stresses plants post-application. One such type of stress is changes to water availability as tissues associated with plant-water relations are incapacitated. Size and shape of pollen grains depend on moisture content, and a higher moisture content is associated with higher rate of germination (Van Hout and Katz, 2004; Pacini et al., 2006). Reduced water content of pollen grains due to GBH-induced water stress potentially contributed to smaller pollen grains, increased variation of pollen shape, proportion of underdeveloped pollen, and reduced viability found in our rose plants. The difference in viability between roses grown in the greenhouse and those in their natural habitat (operational areas) could be further explained by the stress that the plants are under from being potted and kept in a greenhouse, namely, changes in heat and water regime from what would be experienced by the plant growing in its natural conditions. Warmer-than-naturally occurring greenhouse conditions are documented to produce low pollen germination and fruit set in several species (Abdul-Baki and Stommel, 1995; Higuchi et al., 1998).

Our study reveals that the sublethal morphological effects of GBH application extend from most studies on agricultural plants, to treatments applied in natural forest settings, and that the morphological changes observed extend well beyond the season of application. Studies documenting the effects of GBH on plant reproductive morphology have been, to date, conducted directly after applications on agriculturally relevant plants, such as *I. purpurea* (Baucom et al., 2008) and *Gossypium hirsutum* (Pline et al., 2002; Yasuor et al., 2007). We note the vast difference in timeline of residue persistence and morphological deformation in forested environments likely due to the phenological differences when compared to the existing studies on agriculturally grown plants. For example, observations of low-dose GBH applications in agriculturally relevant plants revealed that GBH inhibited dehiscence in flowers that opened directly after the crop plants were sprayed with GBH (Pline et al., 2002; Thomas et al., 2004; Yasuor et al., 2007; Baucom et al., 2008). We also found non-dehiscent and partially dehisced anthers in the treated forest cutblocks; however, the major difference between these studies and ours is that, in our study, we continue to see the impacts of GBH on anther dehiscence in roses one-year post-spray. The flowers we sampled were produced a whole year after the plants were exposed to GBH, indicating that these plants stored GBH residue over the dormant season and then translocated the residues to new tissues the following year, which still caused deformation to occur. In another example, by 4 weeks post-application, pollen viability in agricultural cotton plants was noted to have recovered and GBH appeared to have no effect (Pline et al., 2003b). This is contrary to what we found in prickly rose, which showed treatment-related morphological affects 52 weeks after GBH application. Individuals from the GBH treated sites had glyphosate residue present within reproductive tissues two-years after applications took place, indicating that GBH may potentially have a continued impact on pollen viability. The half-life of glyphosate in plant tissue varies substantially between species and tissue type (Feng and Thompson, 1990; Wood, 2019; Sesin et al., 2021) and is also reported to potentially vary by climatic condition, although more research is required to elucidate that phenomena (Botten et al., 2021). Roy et al. (1989) studied perennial forest plants and reported that glyphosate levels did not dissipate to below 0.01 ppm, but since they only studied up until 60 days post-application, we cannot directly compare this result to our findings. Clearly, as expected with half-life trends, low levels of glyphosate are able to persist for an extended period of time. We demonstrate that plants exposed to GBH in northern environments show extended persistence from what has been previously reported, the impacts of which remain to be studied fully.

Since forests largely contain perennial plants, versus agricultural annuals, study of plants in this alternate ecosystem is of importance, especially given the global decline in natural forest biodiversity (Nischke, 2008; Boutin et al., 2009; Watson et al., 2018). It is critical to fully understand how long GBH-induced change will impact natural forested environments, as these plants are vital food sources for a plethora of wildlife. Our research could be used to define and predict the period of recovery from GBH-related stress. Moreover, we add to the existing literature by describing specific impacts to reproductive morphology in perennial forest plants. The duration between GBH application and sampling, and the degree of morphological changes at the point of sampling, can likely be used for predicting glyphosate residue remaining in the plant and the degree of morphological deformation to be expected.

## Conclusion

It is clear that GBH causes morphological deformation in the reproductive morphology of *R. acicularis*, and that petal color, petal shape, pollen viability, pollen size and shape, and anther development are affected. We found these deformations one year after applications took place, in newly grown tissues, indicating that the GBH persisted in the tissues over the dormant season, unlike the findings in agricultural studies. We also found low levels of persistent GBH residues in flowers of *R. acicularis* 2 years after applications, indicating that residues could continue to have an effect long after applications. Due to the extensive use of GBH in forestry operations and off-target plants receiving application, it is important to understand these effects and interactions, and how these differ from the existing literature based on agricultural systems. Changes in reproductive morphology could have wide-ranging impacts on pollinator species, foraging animals, people who harvest from understory plants, and the overall health of the forest. Further research is required to fully understand the effects of GBH on forest understory plant reproduction. We hope this research will inform forest ecologists and managers and motivate the re-evaluation of current practices to minimize the effects of GBH on wild plants.

## Data Availability Statement

The raw data supporting the conclusions of this article will be made available by the authors, without undue reservation.

## Author Contributions

AG collected the data, conducted experimentation, performed laboratory and statistical analysis, and wrote the report. LW collected the data, conducted some statistical analysis, revised and edited the report, created figures, and supervised the study. All authors contributed to the article and approved the submitted version.

### Conflict of Interest

The authors declare that the research was conducted in the absence of any commercial or financial relationships that could be construed as a potential conflict of interest.

## References

[ref1] Abdul-Baki A. A. Stommel J. R. (1995). Pollen viability and fruit set of tomato genotypes under optimum and high-temperature regimes. HortScience 30, 115–117. 10.21273/HORTSCI.30.1.115

[ref2] Baucom R. S. Mauricio R. Chang S. M. (2008). Glyphosate induces transient male sterility in *Ipomoea purpurea*. Botany 86, 587–594. 10.1139/B08-035

[ref26] Bayer Cropscience Inc. (2020). VisionMAX Silviculture Herbicide commercial solution label. Bayer Cropscience Inc., Calgary, Alberta. Available at: https://www.environmentalscience.bayer.ca/-/media/prfcanada/product-labels/visionmax_label_2020_en.ashx (Accessed June 2, 2021).

[ref3] Belz R. G. Duke S. O. (2014). Herbicides and plant hormones. Pest Manag. Sci. 70, 698–707. 10.1002/ps.3726, PMID: 24446388

[ref4] Blackburn L. G. Boutin C. (2003). Subtle effects of herbicide use in the context of genetically modified crops: a case study with glyphosate (roundup^®^). Ecotoxicology 12, 271–285. 10.1023/A:1022515129526, PMID: 12739874

[ref5] Boateng J. (2002). Herbicide Field Handbook (Revised). British Columbia Ministry of Forests, Forest Practices Branch. FRDA handbook, ISSN 0835–1929; 006 [Rev.].

[ref6] Botten N. Wood L. J. Werner J. (2021). Glyphosate remains in forest plant tissues for a decade or more. For. Ecol. Manage. 10.1016/j.foreco.2021.119259 (in press).

[ref8] Boutin S. Haughlnd D. L. Schieck J. Herbers J. Bayne E. (2009). A new approach to forest biodiversity monitoring in Canada. For. Ecol. Manag. 258, S168–S175. 10.1016/j.foreco.2009.08.024

[ref7] Boutin C. Strandberg B. Carpenter D. Mathiassen S. K. Thomas P. J. (2014). Herbicide impact on non-target plant reproduction: what are the toxicological and ecological implications? Environ. Pollut. 185, 295–306. 10.1016/j.envpol.2013.10.009, PMID: 24316067

[ref9] Brewbaker J. L. Kwack B. H. (1963). The essential role of calcium ion in pollen germination and pollen tube growth. Am. J. Bot. 50, 747–858. 10.1002/j.1537-2197.1963.tb06564.x

[ref10] Cederlund H. (2017). Effects of spray drift of glyphosate on nontarget terrestrial plants – a critical review. Environ. Toxicol. Chem. 36, 2879–2886. 10.1002/etc.3925, PMID: 28731230

[ref11] Dupont Y. L. Strandberg B. Damgaard C. (2018). Effects of herbicide and nitrogen fertilizer on non-target plant reproduction and indirect effects on pollination in *Tanacetum vulgare* (Asteraceae). Agric. Ecosyst. Environ. 262, 76–82. 10.1016/j.agee.2018.04.014

[ref12] Erdtman G. (1943). “Pollen and spore morphology,” in An Introduction to Pollen Analysis. ed. Verdoorn F. (Waltham, Massachusetts, U.S.A.: Chronica Botanica Company), 45–54.

[ref13] Feng J. C. Thompson D. G. (1990). Fate of glyphosate in a Canadian forest watershed. 2. Persistence in foliage and soils. J. Agric. Food Chem. 38, 1118–1125. 10.1021/jf00094a046

[ref14] Fukui H. Hirai N. Mori S. Goto K. Toyoda J. Tsukioka J. (2017). Floral fluorescence database. The garden of medicinal plants. Available at: http://labo.kyoto-phu.ac.jp/mpgkpu/ffd.html (Accessed March 31, 2020).

[ref15] Giura M. Nunez J. Chittka L. Menzel R. (1995). Colour preferences of flower-naïve honeybees. J. Comp. Physiol. A 177, 247–259. 10.1007/BF00192415

[ref16] Govindarajulu P. P. (2008). Literature review of impacts of glyphosate herbicide on amphibians: what risks can the silvicultural use of this herbicide pose for amphibians in British Columbia? British Columbia Ministry of Environment, Victoria, BC, Wildlife Report No. R-28.

[ref17] Gray B. (2011). The Boreal Herbal: Wild Food and Medicine Plants of the North. Whitehorse (Yukon): Aroma Borealis Press, 144–147.

[ref18] Griffin J. L. Boudreaux J. M. Miller D. K. (2010). Herbicides as harvest aides. Weed Sci. 58, 355–358. 10.1614/WS-09-108.1

[ref19] Health Canada (2017). Re-evaluation decision: glyphosate. Health Canada Pest Management Regulatory Agency. 1925-1025.

[ref20] Health Canada (2021). Consumer product safety, label search results. Available at: https://pr-rp.hc-sc.gc.ca/ls-re/lbl_detail-eng.php?p_disp_regn=%2714981%27&p_regnum=14981 (Accessed May 18, 2021).

[ref21] Higuchi H. Utsunomiya N. Sakurantani T. (1998). High temperature effects on cherimoya fruit set, growth and development under greenhouse conditions. Sci. Hortic. 77, 23–31. 10.1016/S0304-4238(98)00160-5

[ref001] Huffman J. L. Riggins C. W. Steckel L. E. Tranel P. J. (2016). The EPSPS Pro106Ser substitution solely accounts for glyphosate resistance in a goosegrass (Eleusine indica) population from Tennessee, United States. J. Integr. Agric. 15, 1304–1312.

[ref22] Hunt J. Matute P. (2019). A review of glyphosate use in British Columbia forestry. Project Number: 301013763, FPInnovations. Written for BC Ministry of Forests, Lands, Natural Resource Operations and Rural Development.

[ref23] MacKinnon A. Pojar J. Coupe R. (1992). Plants of Northern British Columbia. 1st *Edn*. Edmonton (Alberta): Lone Pine Publishing.

[ref24] Marles R. J. Clavelle C. Monteleone L. Tays N. Burns D. (2012). Aboriginal Plant Use in Canada’s Northwest Boreal Forest. 1st *Edn*. Edmonton (Alberta): Natural Resources Canada, 238–239.

[ref25] Meyer S. E. (2008). “Rosaceae-Rose family: *Rosa* L,” in Woody Plant Seed Manual. eds. Bonner F. T. Karrfalt R. P. (Washington, DC: US Department of Agriculture, Forest Service), 974–980.

[ref27] Mori S. Fukui H. Oishi M. Sakuma M. Tsukioka J. Goto K.. (2018). Biocommunication between plants and pollinating insects through fluorescence of pollen and anthers. J. Chem. Ecol. 44, 591–600. 10.1007/s10886-018-0958-9 29717395

[ref28] Nischke C. R. (2008). The cumulative effects of resource development on biodiversity and ecological integrity in the peace-Moberly region of Northeast British Columbia, Canada. Biodivers. Conserv. 17, 1715–1740. 10.1007/s10531-008-9376-6

[ref29] Pacini E. Guarnieri M. Nepi M. (2006). Pollen carbohydrates and water content during development, presentation, and dispersal: a short review. Protoplasma 228, 73–77. 10.1007/s00709-006-0169-z, PMID: 16937057

[ref30] Pline W. A. Edmisten K. L. Oliver T. Wilcut J. W. Wells R. Allen N. S. (2003a). Use of digital image analysis, viability stains, and germination assays to estimate conventional and glyphosate-resistant cotton pollen viability. Crop Sci. 42, 2193–2200. 10.2135/cropsci2002.2193

[ref31] Pline W. A. Edmisten K. L. Wilcut J. W. Wells R. Thomas J. (2002). Reproductive abnormalities in glyphosate-resistant cotton caused by lower CP4-EPSPS levels in the male reproductive tissue. Weed Sci. 50, 438–447. 10.1614/0043-1745(2002)050[0438:RAIGRC]2.0.CO;2

[ref32] Pline W. A. Edmisten K. L. Wilcut J. W. Wells R. Thomas J. (2003b). Glyphosate-induced reductions in pollen viability and seed set in glyphosate-resistant cotton and attempted remediation by gibberellic acid (GA3). Weed Sci. 51, 19–27. 10.1614/0043-1745(2003)051[0019:GIRIPV]2.0.CO;2

[ref33] Potts S. G. Biesmeijer J. C. Kremen C. Neumann P. Schweiger O. Kunin W. E. (2010). Global pollinator declines: trends, impacts, and drivers. Trends Ecol. Evol. 25, 345–353. 10.1016/j.tree.2010.01.007, PMID: 20188434

[ref34] Roy D. N. Konar S. K. Banerjee S. Charles D. A. Thompson D. G. Prasad R. (1989). Uptake and persistence of the herbicide glyphosate (vision^®^) in fruit of wild blueberry and red raspberry. Can. J. For. Res. 19, 842–847. 10.1139/x89-128

[ref35] Sammons R. D. Gaines T. A. (2014). Glyphosate resistance: state of knowledge. Pest Manage. Sci. 70, 1367–1377. 10.1002/ps.3743, PMID: 25180399 PMC4260172

[ref36] Sesin V. Davy C. M. Dorken M. E. Gilbert J. M. Freeland J. R. (2021). Variation in glyphosate effects and accumulation in emergent macrophytes. Manage. Biol. Invasions 12, 66–84. 10.3391/mbi.2021.12.1.05

[ref37] Thomas W. E. Pline-Srnic W. A. Thomas J. F. Edmisten K. L. Wilcut J. W. (2004). Glyphosate negatively affects pollen viability but not pollination and seed set in glyphosate-resistant corn. Weed Sci. 52, 725–734. 10.1614/WS-03-134R

[ref39] Van Hout R. Katz J. (2004). A method for measuring the density of irregularly shaped biological aerosols such as pollen. Aerosol Sci. 35, 1369–1384. 10.1016/j.jaerosci.2004.05.008

[ref40] Watson J. E. M. Evans T. Venter O.. (2018). The exceptional value of intact forest ecosystems. Nat. Ecol. Evol. 2, 599–610. 10.1038/s41559-018-0490-x 29483681

[ref41] Wood L. J. (2019). The presence of glyphosate in forest plants with different life strategies one year after application. Can. J. For. Res. 49, 586–594. 10.1139/cjfr-2018-0331

[ref42] Wronska-Pilarek D. Jagodzinski A. M. (2011). Systematic importance of pollen morphological features of selected species from the genus *Rosa* (Rosaceae). Plant Syst. Evol. 295, 55–72. 10.1007/s00606-011-0462-y

[ref43] Yasuor H. Riov J. Rubin B. (2007). Glyphosate-induced male sterility in glyphosate-resistant cotton (*Gossypium hirsutum* L.) is associated with inhibition of anther dehiscence and reduced pollen viability. Crop Prot. 26, 363–369. 10.1016/j.cropro.2005.06.015

[ref44] Young J. Hawley A. (2004). Plants and Medicines of Sophie Thomas: Based on the Traditional Knowledge of Sophie Thomas, Sai’kuz Elder and Healer. 3rd *Edn*. Columbia: University of Northern British, 48–49.

[ref45] Zabalza A. Orcaray L. Fernández-Escalada M. Zulet-González A. Mercedes R. (2017). The pattern of shikimate pathway and phenylpropanoids after inhibition by glyphosate or quinate feeding in pea roots. Pestic. Biochem. Physiol. 141, 96–102. 10.1016/j.pestbp.2016.12.005, PMID: 28911748

